# Supplementary figures and images for: Global epidemiological and genetic characteristics of carbapenem-resistant Escherichia coli carrying blaIMP
Source: Microbiol Spectr. 2025 Dec 5;14(1):e03244-25. doi: 10.1128/spectrum.03244-25 (PMC12772345; doi:10.1128/spectrum.03244-25)

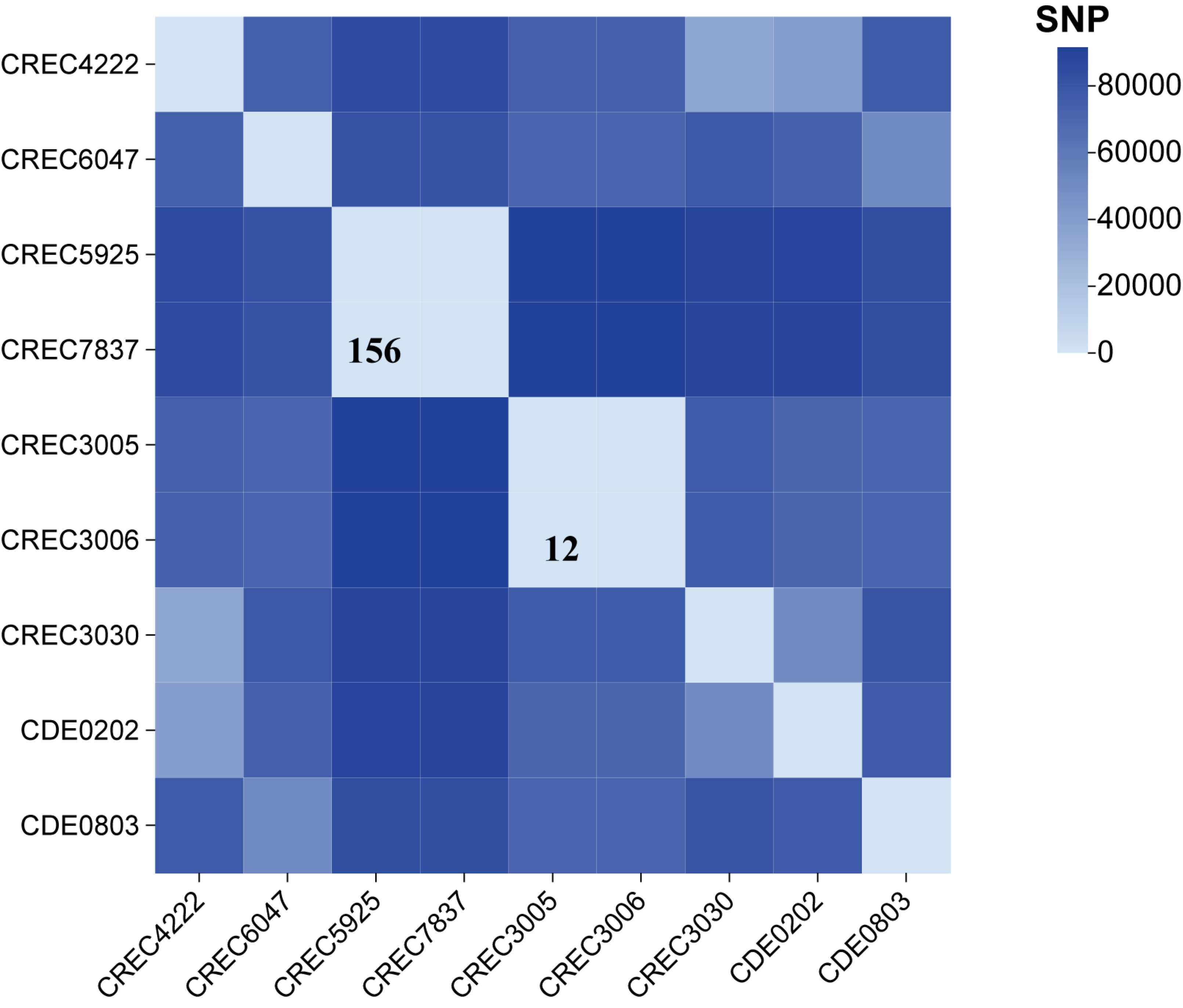

Supplement: Figure S1 — Fig. S1: Cluster analysis of nine blaIMP-positive CRECs based on single nucleotide polymorphisms (SNP). [file spectrum.03244-25-s0001.tif]

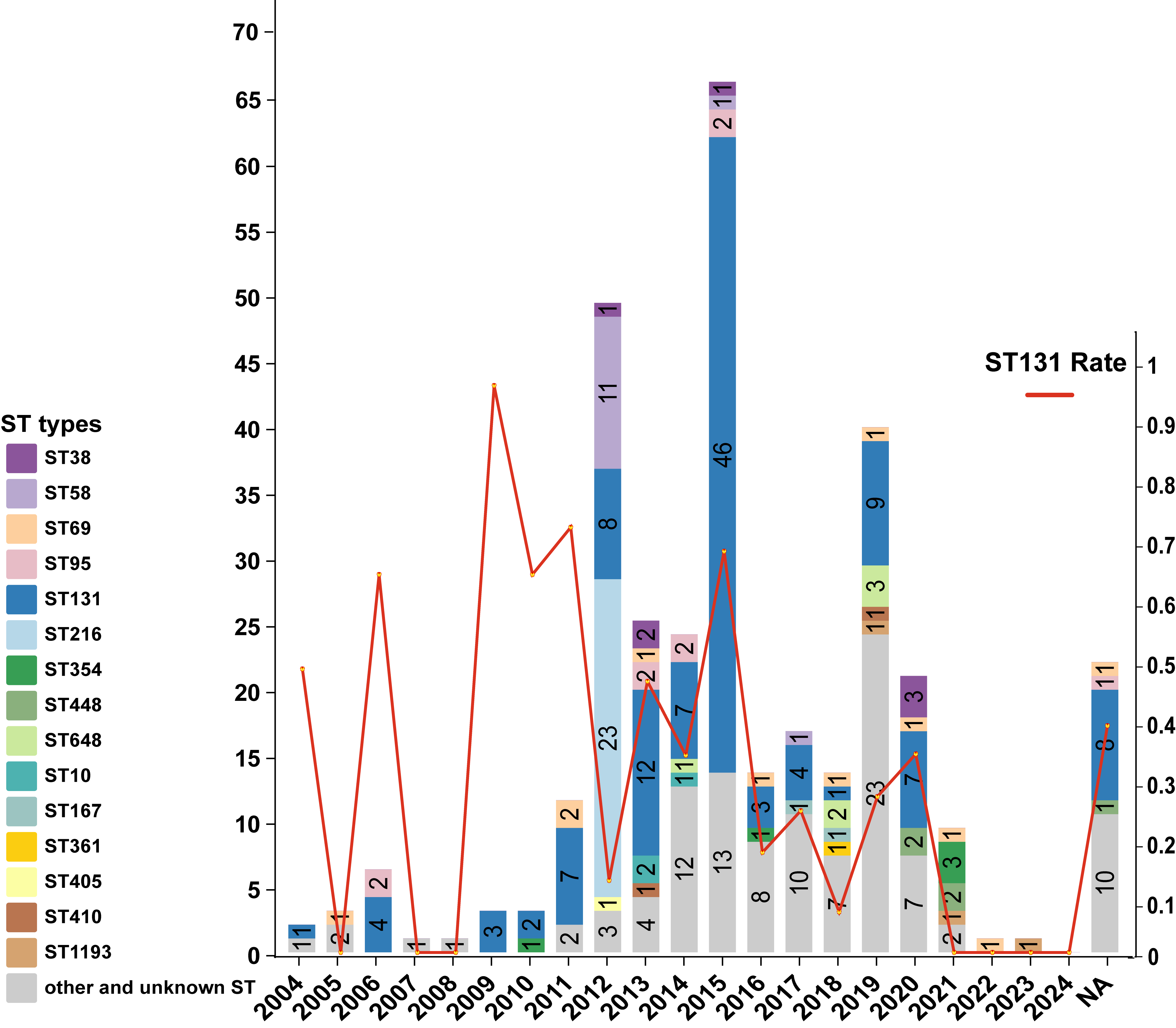

Supplement: Figure S2 — Fig. S2: Bar graph of the number of some STs over time. [file spectrum.03244-25-s0002.tif]

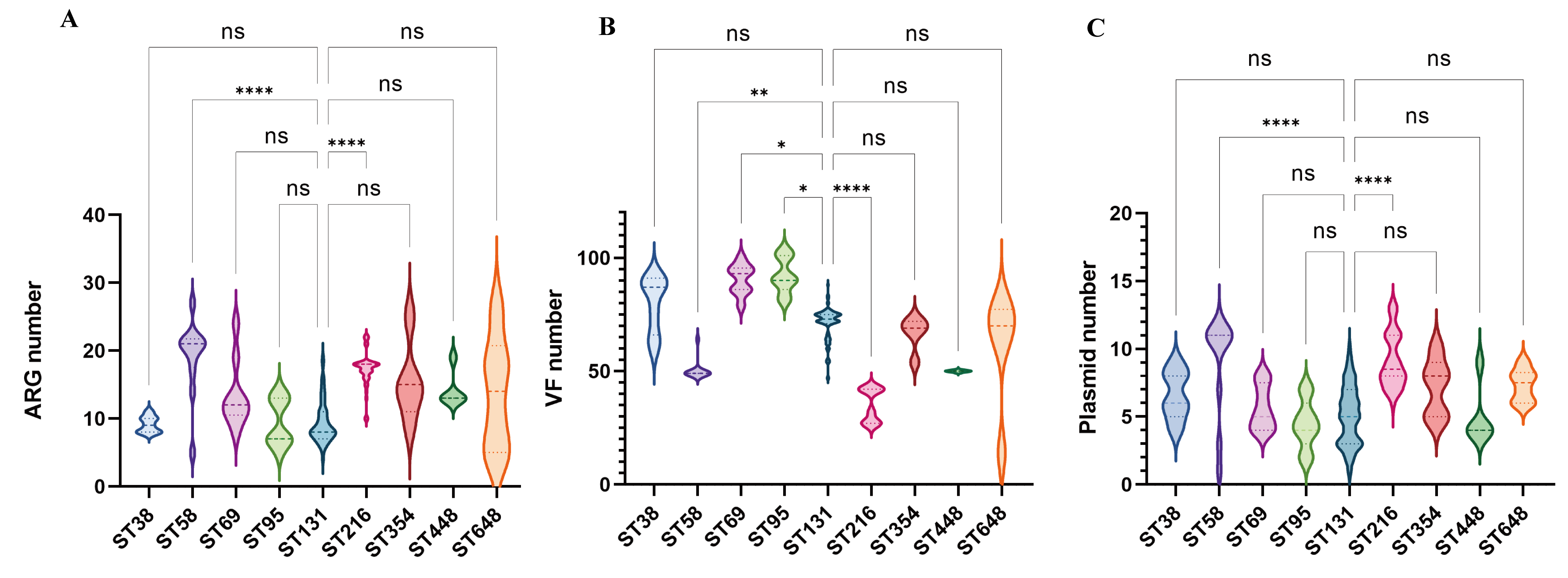

Supplement: Figure S3 — Fig. S3: A correlation analysis between ST types and the number of resistance genes, virulence genes. [file spectrum.03244-25-s0003.tif]
