# Supplementary material for: Global epidemiological and genetic characteristics of carbapenem-resistant Escherichia coli carrying blaIMP
Source: Microbiol Spectr. 2025 Dec 5;14(1):e03244-25. doi: 10.1128/spectrum.03244-25 (PMC12772345; doi:10.1128/spectrum.03244-25)
Supplement: Figure S1 to S3 legends — Fig. S1: Cluster analysis of nine blaIMP-positive CRECs based on single nucleotide polymorphisms (SNP). Fig. S2: Bar graph of the number of some STs over time. Fig. S3: A correlation analysis between ST types and the number of resistance genes, virulence genes. [file spectrum.03244-25-s0004.docx]

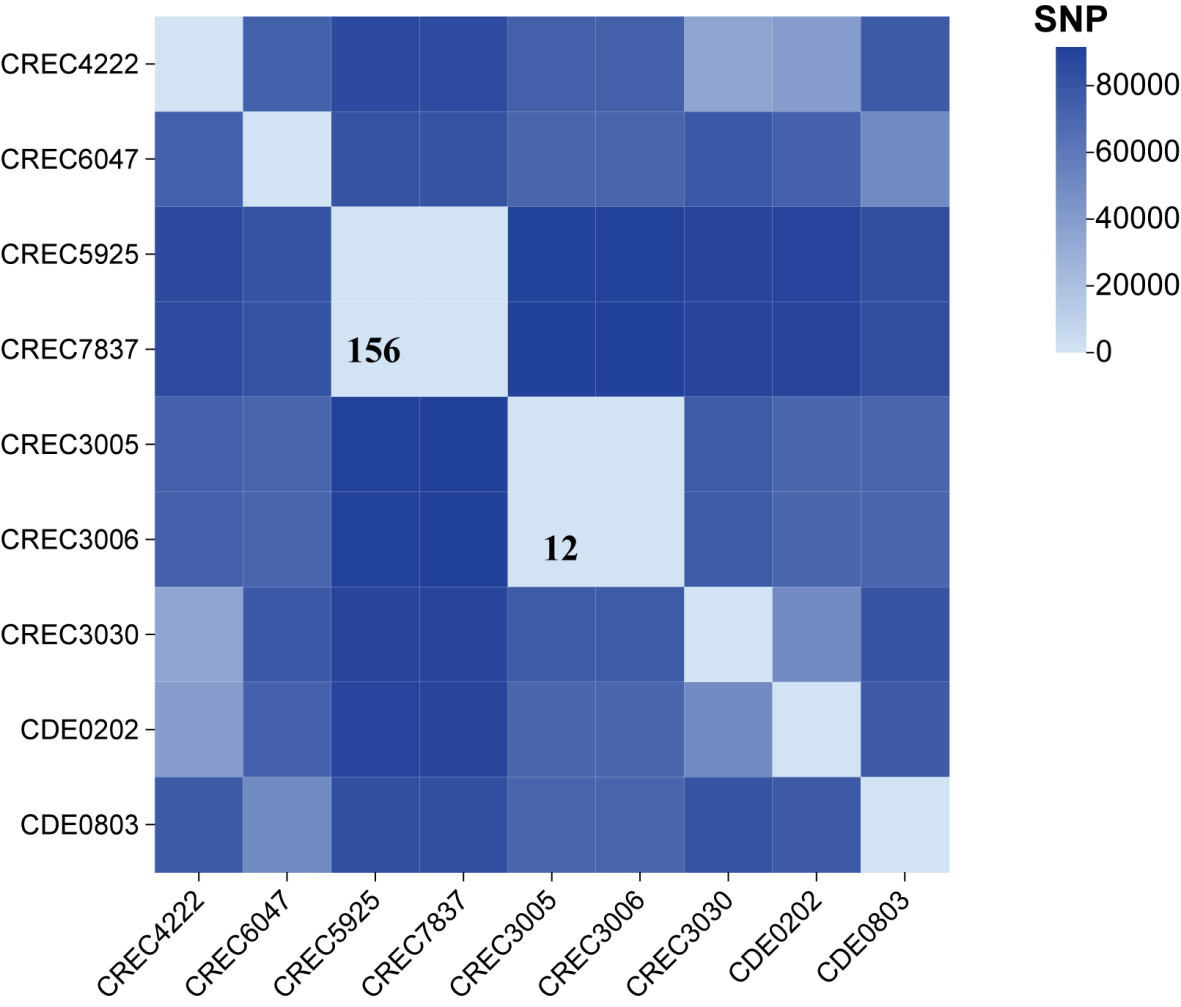


**Figure S1.** **Cluster analysis of nine *bla*_IMP_-positive CRECs based on single nucleotide polymorphisms (SNP).** The SNP-based matrix obtained from Snippy was visualized using Chiplot.


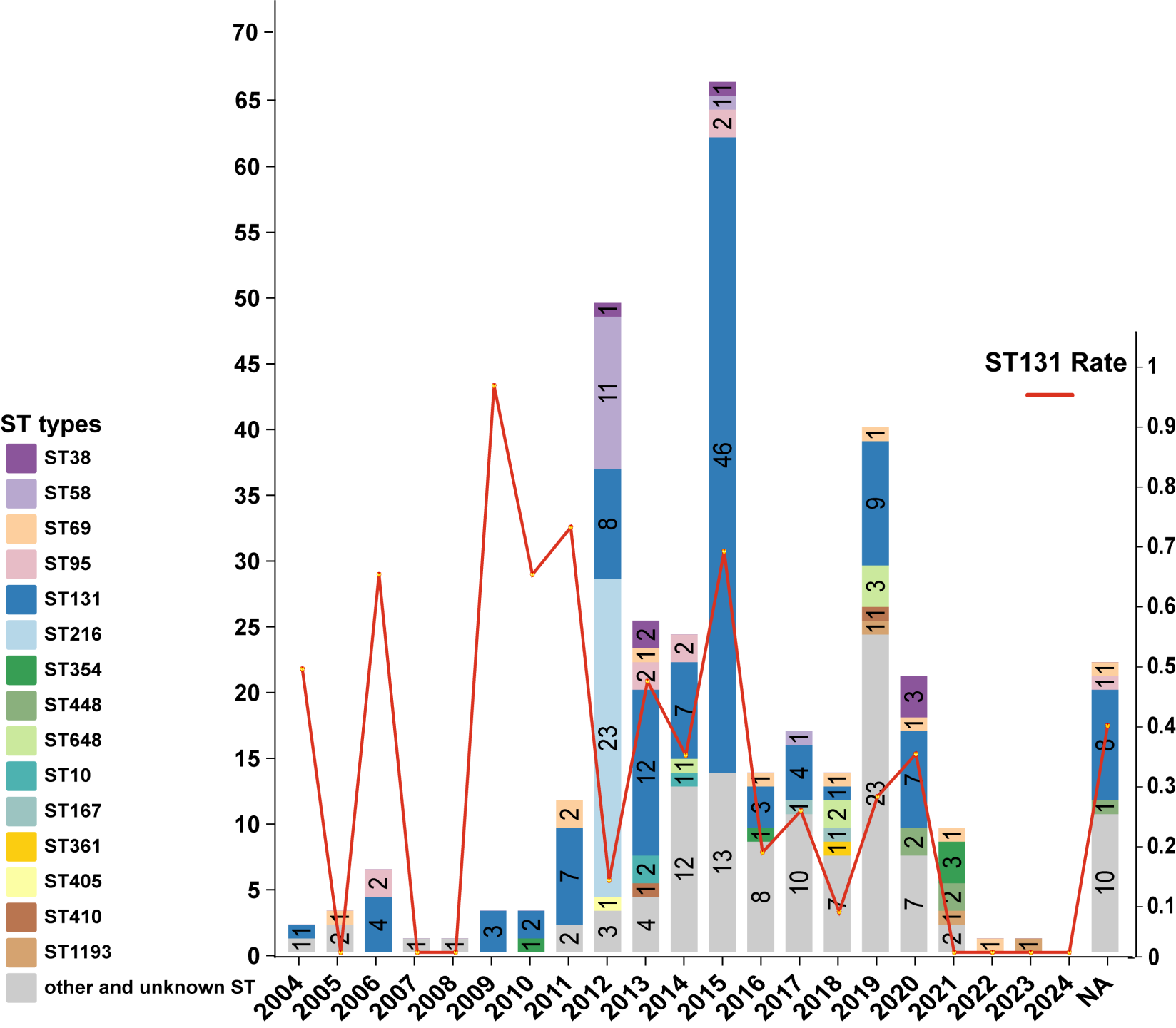


**Figure S2. Bar graph of the number of some STs over time.** The horizontal axis represented the time of *bla*_IMP_-positive CRECs isolation, and the left vertical axis represented the number of strains with different STs. The right vertical axis represented the percentage of strains with ST131.

**
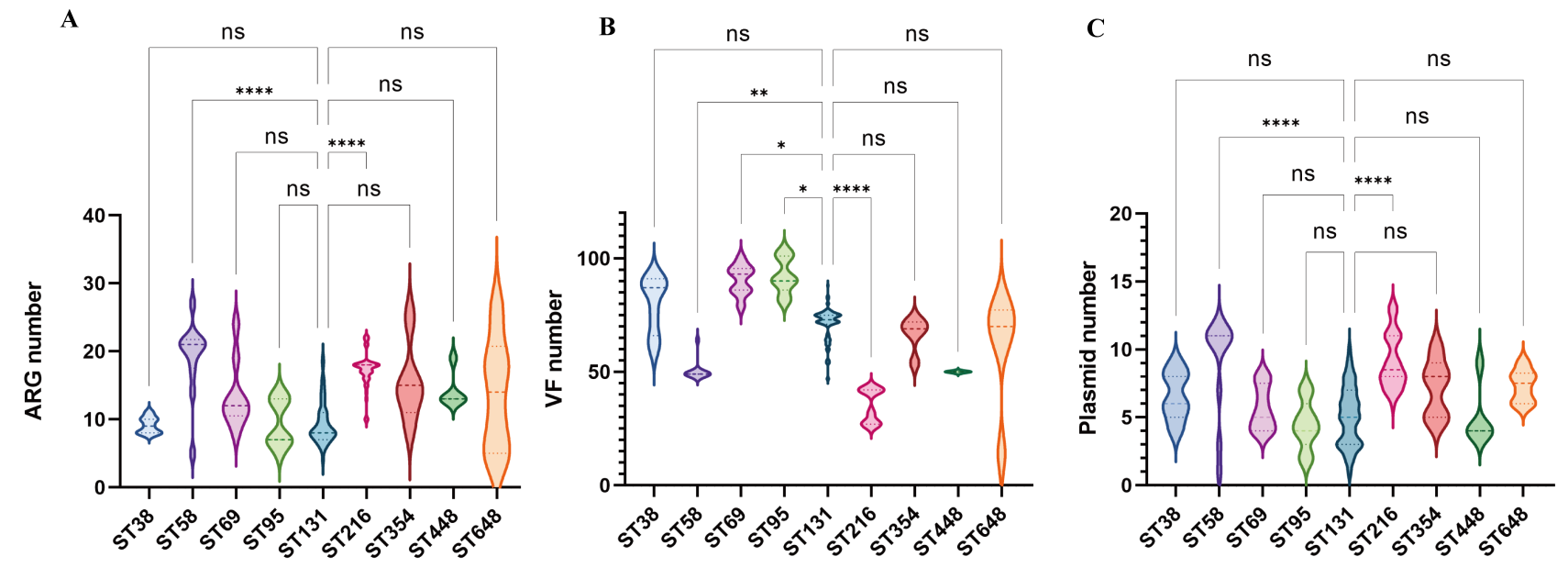
**

**Figure S3. A correlation analysis between ST types and the number of resistance genes, virulence genes. (A)** ARG numbers in different STs *bla*_IMP_-positive CRECs. The horizontal axis represents the different STs, and the vertical axis represents the number of ARGs. **(B)** VF numbers in different STs *bla*_IMP_-positive CRECs. **(C)** Plasmid numbers in different STs *bla*_IMP_-positive CRECs. ns >0.05, *p <0.05; **p <0.01; ***p <0.001; ****p <0.0001 (Kruskal-Wallis test).
